# Quantification of a serum titin fragment reflects dystrophin restoration in mdx mice and disease severity in patients with dystrophinopathies

**DOI:** 10.1177/22143602261453984

**Published:** 2026-05-20

**Authors:** Camilla Johansson, Jessica F. Boehler, Kristy J. Brown, Albert Jiménez-Requena, Andreas Hober, Fredrik Edfors, Åsa Sivertsson, Gabriella Hober, Zaïda Koeks, Nienke M. van de Velde, Mathias Uhlén, Hanna Tegel, Erik H. Niks, Pietro Spitali, Cristina Al-Khalili Szigyarto

**Affiliations:** 1 Department of Protein Science, School of Chemistry, Biotechnology and Health, 7655 KTH Royal Institute of Technology, Stockholm, Sweden; 2 688351 Solid Biosciences Inc, Charlestown, MA, USA; 3 Somite Therapeutics, Brighton, MA, USA; 4 Science for Life Laboratory, School of Chemistry, Biology and Health, 7655 KTH Royal Institute of Technology, Stockholm, Sweden; 5 Department of Neurology, 4501 Leiden University Medical Center, Leiden, The Netherlands; 6 Department of Human Genetics, 4501 Leiden University Medical Center, Leiden, The Netherlands

**Keywords:** duchenne muscular dystrophy, titin, sandwich immunoassay, micro-dystrophin therapy, protein biomarker, becker muscular dystrophy

## Abstract

Increased dystrophin expression in muscle has been accepted as a surrogate endpoint for accelerated approval of gene therapies within Duchenne Muscular Dystrophy (DMD). Previous studies identified an N-terminal titin fragment in urine as a biomarker suitable for monitoring disease progression and therapeutic interventions. Plasma levels of a titin fragment comprising aa 2,229–2,352 were found to negatively correlate with dystrophin expression in *mdx* mice treated with AAV9-CK8-μDys5 micro-dystrophin gene therapy. In this study, we sought to investigate which titin fragments can be detected in serum from patients with DMD and Becker muscular dystrophy (BMD). Using bottom-up selected reaction monitoring tandem mass spectrometry, we identified several serum peptides originating from the central region of titin. Six monospecific antibodies targeting this fragment could recognise a proteolytic titin product at 100 kDa, present in serum from DMD and BMD patients, but not healthy controls. This fragment showed a steeper age-related decline in patients with DMD compared to those with BMD. We further developed a quantitative sandwich immunoassay and showed that the concentrations of this fragment were between 130 and 2,390 pM in DMD patients and 110 and 3,970 pM in BMD patients. The same assay showed titin concentration, between 170 and 990 pM in plasma from treated *mdx* mice, that had a significant Pearson correlation of -0.76 (*P*=1.53e−08) with (micro)dystrophin expression in quadriceps. If the negative correlation with dystrophin expression in muscle is also confirmed in DMD patients treated with micro-dystrophin therapies, this serum titin fragment may serve as a potential pharmacodynamic biomarker.

## 1. Introduction

Duchenne muscular dystrophy (DMD) is a rare, X-linked genetic disorder caused by low or absent dystrophin expression in muscle tissue.^1,2^ Due to the lack of dystrophin expression, patients have progressive and fatal muscle degradation, loss of ambulation, myocardial dysfunction, progressive loss of respiratory function and, in a significant proportion of patients, cognitive impairments.^3^ One therapeutic approach to treat DMD includes micro-dystrophin gene therapy, which aims to address disease progression by delivering a shortened but functionally conserved micro-dystrophin gene into muscle tissues through adeno-associated viral vectors.^2^ The hypothesis of adequate muscle-protection from partial expression of truncated dystrophin proteins is supported by studies of patients with Becker muscular dystrophy (BMD) who, due to in-frame mutations in the *DMD* gene, experience a milder phenotype than DMD patients. In clinical trials evaluating approaches to augment dystrophin or provide micro-dystrophin, quantification of these proteins is performed using muscle biopsies collected at post-treatment timepoints,^4^ which are limited in size and frequency of collection and introduce risks of infection and scarring.^5^ We hypothesise that blood biomarkers may allow for minimally invasive characterisation of changes in DMD disease progression that could provide a less invasive supplement or alternative to muscle biopsies to evaluate the effect of treatment over time.^6,7^

In a previous study, we identified that an antibody raised against amino acid sequence 2,229–2,352 of skeletal muscle titin (TTN, Ensembl ID: ENSP00000467141) could recognise a protein or peptide, which was more abundant in *mdx* mice compared to wild-type mice and had a strong correlation to percentage of micro-dystrophin expression in muscle tissue upon treatment of *mdx* mice with a micro-dystrophin gene therapy.^8^ Similarly, the levels of TTN were shown to decrease in patients treated with the exon 44-skipping drug, brogidirsen (NS-089/NCNP-02).^9^ Furthermore, different serum TTN abundance was detected in DMD patients compared to BMD patients, but not compared to healthy controls.^8^ TTN is the largest protein in the human body and expressed as a 3.3–3.7 MDa protein in striated muscle as part of the sarcomere complex, where it is directly involved in muscle contraction.^10^ In dystrophic tissue, calcium homeostasis is altered,^11^ which causes activation of calcium-dependent proteases, such as calpain. It is believed that calpain, among other proteases, initiates the degradation of TTN.^12^ The resulting proteolytic fragments enter the bloodstream, and some fragments, get cleared through the kidneys into urine.^10,13–16^ An N-terminal fragment of TTN was detected in both *mdx* mice and DMD patient urine,^14^ but with fragments of slightly different sizes between the two species. Other studies identified a 170 kDa fragment of TTN in serum from young DMD patients,^17^ which possibly represents the central region of TTN.

In this study, we set out to explore novel proteolytic TTN fragments in serum from patients with DMD and BMD and explore if any of these regions could be good candidates for monitoring dystrophin rescue during micro-dystrophin gene therapies. In order to explore the prevalence of proteolytic fragments of TTN in serum, we employed both a selected reaction monitoring (SRM) tandem mass spectrometry strategy and produced new monospecific antibodies towards six TTN regions of interest and assessed their binding to titin through Western blot, in-gel digest LC-MS/MS and multiplex immunoassays. Finally, we developed a quantitative sandwich immunoassay towards a central region of TTN and demonstrated that it could detect TTN in both human serum and mouse plasma, and demonstrated correlation with (micro)dystrophin expression in *mdx* mice.

## 2. Materials and methods

### 2.1. Ethics

This study was performed according to the Declaration of Helsinki and approved by the LUMC Commissie Medische Ethiek (protocol NL50171.058.14) and Regionala etikprövningsnämnden Stockholm, Sweden (ref. 2018/1859-31/1). The animal study was conducted at a laboratory operating in compliance with oversight from an Institutional Animal Care and Use Committee (IACUC), accredited by the Association for the Assessment and Accreditation of Laboratory Animal Care, holding the Animal Welfare Assurance and registered with the United States Department of Agriculture.

### 2.2. Patient natural history study design

BMD patients (n=34) were recruited via the Dutch Dystrophinopathy Database for a 4-year prospective natural history study conducted at the LUMC.^18^ DMD patients (n=19) and healthy controls (n=9) were recruited via LUMC. Both disease groups and healthy individuals comprised only males. Serum samples from DMD and BMD patients were donated annually and prepared as described by van de Velde et al. 2023*.*^18^ BMD patients were also asked to consent to and donate a tibialis anterior (TA) biopsy for quantification of dystrophin expression^4^(Table S1). Muscle biopsies were obtained using a conchotome and were immediately snap-frozen in liquid nitrogen for storage at −80 °C. To prepare protein lysates, approximately 5–10 mg of muscle tissue was processed, and dystrophin levels were quantified using a Capillary Western immunoassay as described by Koeks et al. ^4^This method employed the mouse monoclonal antibody Mandys106^19^ along with a rabbit monoclonal anti-dystrophin antibody. A six-point calibration curve was created using a healthy muscle reference sample. Dystrophin and α-actinin signals were reported as a percentage of control to produce muscle content-corrected values.

### 2.3. Targeted LC-MS/MS of serum collected within the natural history study

Serum samples were prepared for LC-MS/MS analysis.^20^ In short, four microliters of heavy-labelled standards were dried and subsequently mixed with 10 µl serum diluted 1:10 in denaturing buffer (4.5 M urea, 10 mM TCEP, 1xPBS) as previously described.^21^ Samples were treated with chloroacetamide, adjusted to 0.5 M urea in 1xPBS and incubated overnight with 1.2 µg trypsin (Sigma Aldrich, St Louis, MO, USA). Following quenching in formic acid (FA), samples were desalted as previously described^22^ and resuspended in 0.1% FA. Approximately 10 µg of peptides were loaded onto an Ultimate 3000 LC-system (Thermo Fisher Scientific, Waltham, MA, USA) equipped with an Acclaim PepMap 100 trap column (PN 160454, particle size: 5 μm, pore size: 100 Å, 0.3 mm x 5 mm, Thermo Fisher Scientific) and a 15 cm EasySpray analytical column (PN ES802A rev.2, particle size: 2 μm, pore size: 100Å, 150 μm x 15 cm, Thermo Fisher Scientific) and connected to a TSQ Altis (Thermo Fisher) mass spectrometer operating in a scheduled SRM mode with a cycle time of 0.5 seconds. The identified TTN peptides and monitored transitions are specified in Table S2 and Table S3. The peptides were eluted from the LC-system using a linear gradient with a flow rate of 3 µl/min and a total method time of 35 min. The mobile phase consisted of solvent A (3% acetonitrile (ACN), 0.1% FA) and solvent B (95% ACN, 0.1% FA) and the gradient specifications are described in Table S4. All raw files were analysed in Skyline-daily (v. 19.1.1.309 (1dc16c97a)).^23^

### 2.4. Antigen design and antibody production

Protein Epitope Signature Tags (PrESTs) were designed and produced according to previously published protocols to be used as antigens.^24–26^ In short, antigen DNAs were designed to cover a 200 amino acid long region of TTN showing the lowest homology to all other human proteins as determined by a homology search algorithm. The constructs were synthesised (Invitrogen™ GeneArt™ Gene Synthesis Service) and cloned into pAff8c vectors (containing a hexahistidine tag and an albumin binding protein, His_6_-ABP, in the N-terminal region) and transformed into the *E. coli* strain BL21 (DE3). Cells were cultivated and harvested as previously described.^25^ The cells were lysed, and the proteins were purified under denaturing conditions by using Immobilised Metal Affinity Chromatography.^26^ Pure proteins in elution buffer (6 M Urea, 50 mM NaH_2_PO_4_, 100 mM NaCl, 30 mM Acetic acid, 10 mM NaAc, pH 5.0) were diluted in PBS to get a final buffer composition of 1 M Urea, then concentrated to 1 mL using a Vivapore 10/20 ml concentrator (Vivascience, Hannover, Germany).^27^ PrEST concentration was determined using BCA^TM^ Protein Assay Kit (Pierce, Rockford, IL) according to manufacturer’s instructions and purity assessed through SDS-PAGE under reducing conditions using a 26-well Any kD Criterion TGX Precast Midi Protein gel (Bio-Rad, California, USA).

Immunisation of the rabbits was performed by Innovagen AB (Lund, Sweden). In short, one rabbit per antigen was immunised with 100 µg PrEST for primary immunisation, followed by two booster immunisations of 100 µg at 2-week intervals and two booster immunisations of 100 µg at 4-week intervals (four boosters in total). 20 ml serum was collected at week 7 and 10, and antibody production was assessed through ELISA. Final bleed was performed 2 weeks after the fourth booster immunisation. Antibodies were purified as previously described^27^ through His_6_-ABP-depletion followed by purification on PrEST-coupled HiTrap columns (Cytiva, Marlborough, USA) in an ÄKTAxpress system (Cytiva, Marlborough, USA). Purified antibodies were eluted in PBS and mixed 1:1 with sterile 85% glycerol with 1:2000 ProClin^TM^ 300 (Sigma-Aldrich, Darmstadt, Germany), followed by aliquoting and storage at -20°C.

### 2.5. Antibody validation on Western Blot

Antibody binding to titin was assessed on Western blot using 20 µg human skeletal muscle tissue lysate (GeneTex Inc., USA). Cross-reactivity was assessed on Western Blot using a standard panel of samples (RT4 cell lysate, U-251 MG cell lysate, human albumin-depleted plasma, liver and tonsils).^28^ Antibody binding to serum proteins was assessed in serum from three DMD patients, three BMD patients and a pool of healthy controls as follows. 10 µl serum was depleted on High-Select™ HSA/Immunoglobulin Depletion Resin (Thermo Scientific, Massachusetts, USA) according to the manufacturer’s instructions. The eluate was concentrated down to 40 µl using a 3 kDa cut-off Amicon Ultra 0.5 ml spin column (Millipore, Massachusetts, USA). 20 µl of concentrated, depleted serum was mixed with 10 µl 3xRed, heat-treated for 10 min at 70°C and subsequently separated on a 10-well 4-20% Mini-PROTEAN® TGX Stain-Free ^TM^ gel (Bio-Rad, California, USA) at 180V. Separated proteins were transferred to a Trans-Blot Turbo Mini 0.2 µm PVDF membrane (Bio-Rad, California, USA) using a Trans-Blot Turbo transfer system (Bio-Rad, California, USA) at 1.3A for 10 min. Membranes were subsequently blocked in blocking buffer containing 5% low-fat milk (Semper, Stockholm, Sweden) in TBST for 45 min at room temperature. Primary antibodies were diluted 1:200 in 4 ml blocking buffer and incubated with membranes overnight at 4°C, followed by washing with TBST and incubation with goat anti-rabbit IgG HRP (Thermo Fisher, Massachusetts, USA) diluted 1:8,000 in blocking buffer for 45 min at room temperature. Membranes were washed again in TBST, developed using Immobilon Western Chemiluminescent Horse-Radish Peroxidase substrate (Millipore-Sigma) and images captured using a ChemiDoc XRS+ system (Bio-Rad, California, USA).

### 2.6. In-gel digest LC-MS/MS

One DMD serum sample and one commercial normal human serum sample (Invitrogen) were HSA/Immunoglobulin-depleted and concentrated as described above and separated on a 4-20% Mini-PROTEAN® TGX gel (Bio-Rad, California, USA) at 180V. Bands corresponding to regions > 250 kDa, 130-250 kDa and 80-130 kDa were excised from the gel using a scalpel and cut into 1x1 mm pieces. Normal human serum was separated and excised in duplicate. Gel pieces were washed and shrunk in 500 µl neat ACN for 10 min, followed by reduction in 10 mM dithiothreitol, 100 mM ammonium bicarbonate for 30 min at 56°C. Reduced gel pieces were shrunk in 500 µl ACN for 10 min and incubated with 55 mM 2-chloroacetamid 100 mM ammonium bicarbonate for 20 min in the dark. Following incubation, gel pieces were shrunk in ACN and de-stained in 100 µl of 100 mM ammonium bicarbonate/ACN (1:1, vol/vol) for 30 min, shrunk in ACN and digested overnight at 37°C in 50-100 µg trypsin. In-gel digested peptides were extracted in 1:2 (vol/vol) 5 % fFA/ACN for 15 min at 37°C and 600 rpm. Extracts were collected from gel pieces, dried down in a vacuum centrifuge and redissolved in 0.1% FA before cleaning/desalting extracts on C18 tips to remove any residual gel from the extract. 30% of extracted peptides from each band were injected onto an Ultimate 3000 LC-system (Thermo Fisher Scientific, Waltham, MA, USA) equipped with an Acclaim PepMap 100 trap column (75 µm x 2 cm, 3 µm, 100 Å) and an EASY-Spray ion source connected to a Q-exactive HF Hybrid Quadrupole-Orbitrap Mass Spectrometer. The flow rate was 7 μL per min, using 3% ACN, 0.1% FA and 96.9% water as solvent. Peptides were separated using a ES802 EASY-Spray PepMap RSLC C18 Column (75 µm x 25 cm, 2 µm, 100 Å) at flow rate of 0.7 µL per minute and a 40 minutes linear gradient from 1% to 32% with 95% ACN, 0.1% FA and 4.9% water as secondary solvent and analyzed using one full scan (resolution 60,000 at 200 m/z, mass range 300-1200 m/z) followed by 10 MS2 DDA scans with the 10 most abundant peptides (resolution 30,000 at 200 m/z with an isolation window of 2.0 m/z). Precursor ions were fragmented with high-energy collision-induced dissociation at an Normalised Collision Energy of 26. Maximum injection time and automatic gain control were set to 205 ms and 1E6 for MS1, and to 105 ms and 2E5 for MS2. The MaxQuant software, version 2.4.13.0, was employed to search the RAW files, using a database consisting of the human proteome (Uniprot id: 9606, 20,446 entries, assessed 2024-01-25). Trypsin was selected as the digestion enzyme, with a maximum of two missed cleavages allowed. Peptides shorter than seven amino acids were excluded, and the maximum peptide mass was set to 4600 Da. Methionine oxidation and N-terminal protein acetylation were set as variable modifications. Cysteine carbamidomethylation was set as a fixed modification. Both peptides and proteins were filtered with a maximum FDR of 0.01. Default settings were maintained for all other parameters within MaxQuant.

### 2.7. Multiplex antibody suspension bead array

Patient serum samples were biotin labelled at 20 µg EZ-link NHS-PEG4-biotin (Thermo Scientific, Massachusetts, USA) for 3 µl serum according to previously established protocols.^29^ Antibodies were coupled to magnetic, colour-coded MagPlex microspheres (Luminex Corp., USA) at a concentration of 3.5 µg antibody per million beads as previously described.^29^ Prior to coupling, antibodies T1, T2, T3, T4, T5 and T6 were first purified on Protein A Dynabeads^TM^ (Invitrogen, Massachusetts, USA) to remove residual rabbit albumin, which had been observed in some of these antibodies (as assessed on SDS-PAGE). In short, 4 µg antibody was diluted to 30 µl in PBST and captured on 20 µl Protein A Dynabeads for 30 min. Captured antibodies were washed twice in 100 µl PBST and eluted in 20 µl acetate, pH 3.0, for 2 min. Eluted antibodies were transferred to a clean tube and neutralised in 10 µl 0.75M Tris, pH 8. Antibodies were left to refold on ice for 60 min, then buffer exchanged to 0.1M MES buffer, pH 4.5, on a 10 kDa cut-off Amicon Ultra 0.5 ml spin column (Millipore, Massachusetts, USA) to remove the Tris buffer, which can interfere with NHS coupling. The whole volume of purified and concentrated antibody was adjusted to 100 µl in 0.1M MES buffer for subsequent coupling to MagPlex beads. Two negative control beads were created, one coated with control IgG from rabbit serum (Sigma-Aldrich, Darmstadt, Germany) and one without protein (hereon referred to as empty bead).

Beads were pooled at equal concentrations to create a suspension bead array (SBA). Labelled serum was diluted to approximately 0.24% in an assay buffer containing 0.5 mg/ml rabbit IgG (Sigma-Aldrich, Darmstadt, Germany), 1:1,000 ProClin^TM^ 300 (Sigma-Aldrich, Darmstadt, Germany) 0.025% (w/v) polyvinylalcohol (Millipore, Massachusetts, USA), 0.04% (w/v) polyvinylpyrrolidone (Millipore, Massachusetts, USA), 0.005% casein in PBST and heat-denatured at 56°C for 30 min. SBA was spiked into each sample at approximately 500 beads/bead ID and incubated overnight at 4°C on a thermo plate shaker, 800 rpm. Following incubation, beads were washed in PBST, visualised using streptavidin R-phycoerythrin as previously described^29^ and analysed on a Luminex 200 instrument with xPONENT software.

### 2.8. Mouse sample collection and study design

To study the effect of micro-dystrophin on plasma biomarkers, plasma was selected across six studies to obtain a wide range of doses, as well as micro-dystrophin expression. In those six studies, five-week-old mdx C57BL/10ScSn-Dmdmdx/J male mice were obtained from The Jackson Laboratory (Bar Harbor, ME, USA). The mice were fed with standard mouse chow and water *ad libitum* and kept under a 12-h:12-h light:dark cycle. The mdx mice were administered an AAV9 vector containing a muscle-specific promoter (CK8) and micro-dystrophin (μDys5), as a single intravenous (IV) bolus after one week of acclimatisation.^30^ Administration was performed at 6 weeks of age (Day 1) at levels of 1.6E12, 7.8E12, 1.6E13, 2.4E13, 5E13, 1E14 and 2E14 vg/kg. These administrations were performed over a variety of different studies and were selected for further analysis based on their micro-dystrophin expression in the skeletal muscle. Blood was collected prior to necropsy on Day 29 for treated mdx mice and was banked as plasma after centrifuging at 2200 ×g for 15 minutes at room temperature within 45 minutes of collection. The plasma samples were transferred to cryovials and maintained on dry ice prior to storage at -80 °C. Since this was a retrospective study, wild-type (C57BL/10ScSn/J) and mdx controls (12 weeks of age) were supplemented to provide disease controls. Following necropsy and cryopreservation, quadricep muscles were analysed using immunofluorescence to quantify the percentage of muscle fibers with membrane-localised micro-dystrophin protein. Isopentane frozen muscles were sectioned (8 microns) and stained for micro-dystrophin using MANEX44A targeting exon 44 (DSHB).^31,8,32^ Dystrophin expression was determined as the percentage of muscle fibers positively stained with anti-dystrophin antibodies MANEX44A on a group of whole quadricep muscles comprising approximately 3,000–10,000 myofiber. Assessment of dystrophin expression was performed by a board-certified pathologist, in 5% increments, with finer resolution (1–4%) used for values below 5%. Further details on the method have been previously published.^32^

### 2.9. Design of a sandwich immunoassay

Anti-titin antibodies were used as either capture reagent coupled to MagPlex beads or as biotin-labelled detection antibodies, as previously described.^33^ Antibodies were used either as capture or detection antibodies or split into two fractions and used as both capture and detection antibodies. Diluted serum samples were denatured and incubated with antibody-coupled MagPlex beads overnight at 4°C, followed by washing to remove residual serum and the unbound proteins. Detection antibodies were added, subsequently to the beads as described previously.^33^ The sandwich assay was visualised by streptavidin R-phycoerythrin prior to analysis.

### 2.10. Statistical analysis

All statistical analyses were performed in R version 4.1.0.^34^ Multiplexed SBA data was normalised using probabilistic quotient normalisation.^35^ Linear mixed effects models were performed using R package *lme4*.^36^ All *P*-values were adjusted using the Benjamini-Hochberg method^37^ to account for multiple hypothesis testing and displayed as false discovery rates (FDR). To evaluate univariate associations, Spearman rank correlations were computed between each of the 30 titin peptides’ normalised MS intensities, titin concentration estimated using the sandwich immunoassay and the percentage of dystrophin expression in the TA of 13 BMD patients. Standard curves in sandwich immunoassay experiments were fitted using four-parameter regression in Excel.

## 3. Results

### 3.1. Tandem mass spectrometry identified peptides from the central region of TTN in patient serum and guided the development of new monospecific anti-TTN antibodies

**Figure 1.**
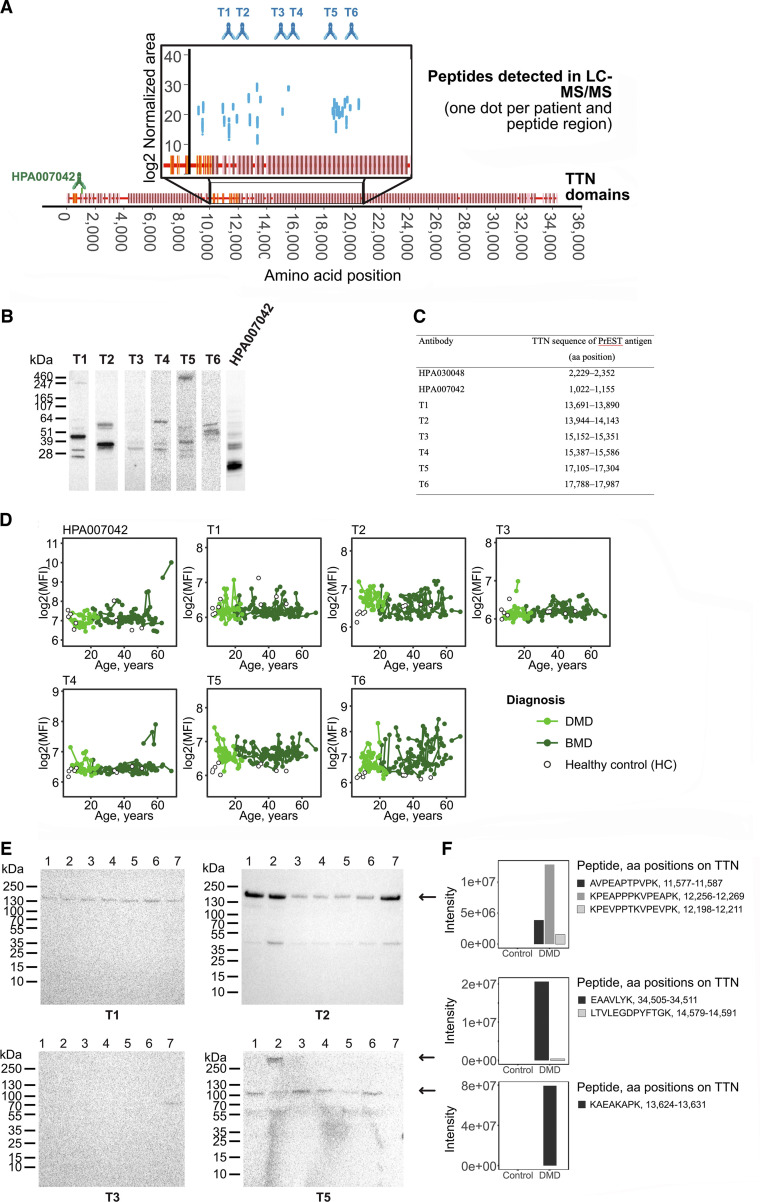
Development of new anti-TTN antibodies based on results from LC-MS/MS detected peptides. (a) Schematic overview of skeletal muscle TTN. Using LC-MS/MS (light blue dots), we identified 31 peptides from central region of TTN in serum from patients with DMD and BMD. Six new monospecific rabbit antibodies (T1, T2, T3, T4, T5 and T6) were raised against central region of TTN. (b) Western blot on healthy human skeletal muscle lysate. Only antibodies T5 recognized bands above 500 kDa (separation limit of gel), possibly corresponding to skeletal muscle TTN (3,500 kDa). (c) Antigen amino acid (aa) sequences used for producing anti-TTN antibodies. Sequences are based on ENSEMBL entry ENSP00000467141. (d) All anti-TTN antibodies were tested on a longitudinal serum cohort with DMD/BMD patients and healthy controls using antibody suspension bead array technology.^2^ Spaghetti plots showing change of TTN fragments with age. Colours indicate patient group. Samples from the same patient are connected. (e) Western blot using patient serum samples identify bands for T5 antibody at 300 kDa and 100 kDa in dystrophinopathy patients. 1: DMD 1. 2: DMD 2. 3: DMD 3. 4. BMD 1. 5: BMD 2. 6: BMD 3. 7: Healthy control pool. (f) In-gel digest LC-MS/MS of bands between 130-250 kDa (top) and 80-130 kDa (bottom) from serum from patient DMD 2 and a commercial healthy control serum pool identified peptides from TTN (Uniprot ID: Q8WZ42) in both bands.

Our previous studies have identified TTN, using the antibody HPA030048 raised against a 124 amino acids (aa) fragment between aa 2,228 and 2,352, which correlates strongly with tissue dystrophin expression in *mdx* mice upon treatment with a micro-dystrophin therapy^38^. The same antibody, however, did not show any clear relationship between dystrophin expression and serum TTN levels in a dystrophinopathy natural history study. In this study, we therefore sought to first elucidate what regions of TTN can be detected in human serum and can potentially be used as a biomarker, and subsequently develop an antibody-based biomarker quantification assay. A study by Hathout et al. identified a 170 kDa large fragment of TTN in serum from DMD patients,^17^ which was hypothesised to map to the central region of the protein. Hence, we focused on the middle part of titin for this project. Thirty-one peptides were identified in a longitudinal serum cohort (Table S1) from patients with either DMD or BMD by using a tandem mass spectrometry approach (Table S2). These peptides are representative of the central region of TTN (light blue dots in Figure 1(a)) spanning from aa 12,198 to aa 21,599 of the skeletal muscle isoform (Ensembl ID ENSP00000467141).

To develop a quantification sandwich immunoassay, six new rabbit monospecific antibodies (blue antibodies in Figure 1(a)) were raised against the central region of TTN using a previously described strategy. Seven PrESTs were produced against different aa sequences of the TTN central region based primarily on *in silico* predictions of antigenicity and sequence homology based on a previously published strategy,^24^ but with preference for overlapping with peptides characterised in the targeted mass spectrometry experiment. These PrESTs, which, apart from the antigen sequence, also contain an albumin binding domain and His-tag, were produced in *E. coli* and subsequently affinity-purified. Six out of seven PrESTs were found to be sufficiently pure (Figure S1) for immunisation in rabbits. Rabbit antisera was affinity purified using a previously published protocol,^28^ resulting in six monospecific antibodies (T1-T6 in Figure 1(b-c) , antigen regions are illustrated in blue in Figure 1(a)). An already existing antibody from the Human Protein Atlas^38^, HPA007042, was also included in the study.

The antibodies produced within this project (T1, T2, T3, T4, T5 and T6), as well as antibody HPA007042, were analysed for binding to TTN through Western Blot on human skeletal muscle lysate (Figure 1(b)). Since TTN has a molecular weight of 3.3-3.7 MDa and the gel has a size limit of 500 kDa, we hypothesised that a band > 500 kDa could correspond to TTN in this setup. Only antibody T5 recognised a band > 500 kDa. Furthermore, the newly produced antibodies were tested against a previously published^28^ standard panel of samples (Figure S2A), which showed low cross-reactivity of antibody T5 to proteins with lower molecular weight in the tested samples.

Using an antibody suspension bead array with all seven anti-TTN antibodies, patient-specific longitudinal signatures were studied. Wilcoxon ranked sum test, and Kruskal-Wallis test on samples at first visit revealed that only antibodies T2, T3, T5 and T6 could reliably discriminate between DMD patients and healthy controls (Table 1, Figure S3) with significant FDR between 4.88E10-02 and 6.28E-04. A linear mixed effects model was used to investigate if there were any differences in trajectories between DMD, BMD and healthy controls. In the model, patient IDs were used as random effects and time from first visit (in years), diagnosis and the interaction between time and diagnosis as fixed effects. Only T5 was found to have a clear time-dependent trajectory in DMD patients and displayed a change in both trajectory and intercept for BMD patients and healthy controls (Table 2, Figure 1(d)) with a significant FDR of 3.25E-02.

**Table 1.** Differences between patient groups at first visit. The differences between the groups were assessed through Wilcoxon rank-sum test and Kruskal-Wallis test. The Benjamini-Hochberg method was used to adjust for multiple hypothesis testing. Adjusted *P*-values are displayed as FDR.

Wilcoxon rank-sum test							Kruskal-Wallis test
Antibody	DMD/BMD log_2_(FC)	DMD/BMD FDR	DMD/HC log_2_(FC)	DMD/HC FDR	BMD/HC log_2_(FC)	BMD/HC FDR	FDR
HPA007042	–0.145	3.77E–01	–0.095	1.00E+00	0.050	7.88E–1	8.94E–03
T1	–0.011	5.70E–01	–0.206	3.11E–02	-0.195	4.69E–2	5.96E–02
T2	0.120	2.66E–01	0.299	2.51E–03	0.179	4.69E–2	5.28E–05
T3	–0.085	6.99E–03	–0.091	4.88E–02	-0.006	7.88E–1	1.89E–02
T4	–0.015	9.73E–01	0.061	2.31E–01	0.075	1.23E–1	2.59E–01
T5	–0.090	3.81E–01	0.364	6.28E–04	0.455	4.25E–8	3.12E–06
T6	–0.139	3.77E–01	0.557	6.28E–04	0.696	4.25E–8	2.16E–06

**Table 2.** A linear mixed effects model identified that T5 and T2 had different trajectory over time between DMD, BMD and healthy controls. T5 was found to correlate with time from first sample in DMD and had a change in time-trajectory between DMD and BMD. While T5 declined with time in DMD, no time-dependent change was observed in BMD. P-values were adjusted for multiple hypothesis testing using the Benjamini-Hochberg method.

Antibody	FDR*				
	*Intercept*	*Time*	*BMD*	*HC*	*Time:BMD*
HPA007042	1.07E–07	8.11E–01	8.27E–01	7.22E–01	2.16E–01
T1	3.15E–40	9.39E–01	9.44E–01	3.56E–02	8.23E–01
T2	2.37E–50	8.11E–01	4.24E–01	8.83E–03	8.23E–01
T3	2.24E–45	1.76E–01	4.24E–01	5.41E–01	3.25E–02
T4	5.74E–38	9.39E–01	8.27E–01	2.70E–01	8.23E–01
T5	6.51E–45	6.82E–03	9.44E–01	1.83E–04	3.25E–02
T6	7.00E–16	8.11E–01	4.88E–01	7.55E–02	5.35E–01

*Significant adjusted p-values for each parameter in the model. *β*_intercept_: fixed effect for intercept in DMD patients. *β*_time_: fixed effect for time trajectory in DMD patients. *β*_BMD_: fixed effect for change in intercept between BMD and DMD patients. *β*_HC_: fixed effect for change in intercept between HC and DMD patients. *β*_Time:BMD_: fixed effect for change in time trajectory between BMD and DMD patients.

Further Western blot analysis of albumin/IgG-depleted serum from three DMD patients, three BMD patients and a control serum pool (Figure 1(e)) revealed that T5 binds a protein of 100 kDa in only DMD and BMD patients but not in healthy controls. Furthermore, the antibody also recognises in the DMD 2 sample, a protein above 250 kDa, most likely representing the full-length TTN. This suggests that T5 binds a patient-specific protein fragment, which is in line with the results observed in the immunoassay. The bands detected by T5 remained patient-specific when repeating the Western blot with a 100 kDa band visible only in the patient pool sample and not in the control sample (Figure S2B). On the other hand, T2, which in the immunoassay appeared more abundant in DMD compared to healthy control, bound to two bands (40 and 150 kDa, respectively), which were present in both DMD, BMD and healthy controls. The T1 and T2 antibodies detect TTN fragments that are less likely to discriminate between DMD and healthy individuals. We further performed in-gel digest LC-MS/MS of the two bands detected by T5, as well as the region which was recognised by T1 and T2 (Figure 1(e-f) bands indicated by arrows), on one DMD serum sample and one healthy control serum sample. Peptides from TTN were detected in two of the three bands (Figure 1(e-f)) in either DMD or healthy controls. The band at > 250 kDa remained inconclusive. For the band at approximately 100 kDa identified by the T5 antibody, TTN peptides were only detected in serum from DMD patients, which aligns with the band being absent in control serum on Western blot. All identified proteins within the target ranges of each excised band are shown in Figure S4. Taken together, this suggests that the T5 antibody binds a fragment of TTN which is patient-specific and discriminative between DMD, BMD and healthy controls, which are the main requirements for a potential gene-therapy monitoring biomarker.

### 3.2. Development of a quantitative sandwich immunoassay against a 100 kDa fragment of TTN, including aa 17105–17304

To confirm the observed behaviour of T5, we sought to develop a quantitative sandwich immunoassay targeting the TTN region aa 17,105–17,304. To develop the sandwich assay different antibody pair combinations, were tested (Figure S5A.). The T5 self-sandwich had the highest signal to background estimated with a 2-fold at 1:25 diluted serum. The sandwich with T5 as capture antibody and T4 as detection antibody had 1.3-fold higher signals than the background. Furthermore, the T5 self-sandwich showed detected TTN in DMD patients in a dose-dependent manner (Figure S5B), with higher levels in a pooled serum sample from ambulant patients compared to non-ambulant patients. T5 also showed low compatibility for dual binding of the target with other antibodies apart from T4. Extensive immunoassay development was undertaken for a T5 self-sandwich (Figure S6). It was demonstrated that the T5 self-sandwich could use its PrEST antigen as a protein standard in calibration curves and spike-in experiments (Figure S6A), but that there was a matrix effect in patient serum compared to analysing PrEST in only buffer. This effect could be mimicked and minimised using the addition of guinea-pig serum into the standard sample at comparable dilutions to diluted patient serum (Figure S6D). Further optimising in terms of sample heat treatment, serum dilution, detection antibody concentration and biotinylation procedure, as well as choice of instrument and signal amplification, resulted in a quantitative assay with a spike-in recovery of 106% ± 19% (Figure S6F). The assay has the LOD of 1.1ng/ml (26 pM TTN), a LLOQ of 1.8 ng/ml (44 pM) and the ULOQ of 200ng/ml (4,988 pM).

**Figure 2.**
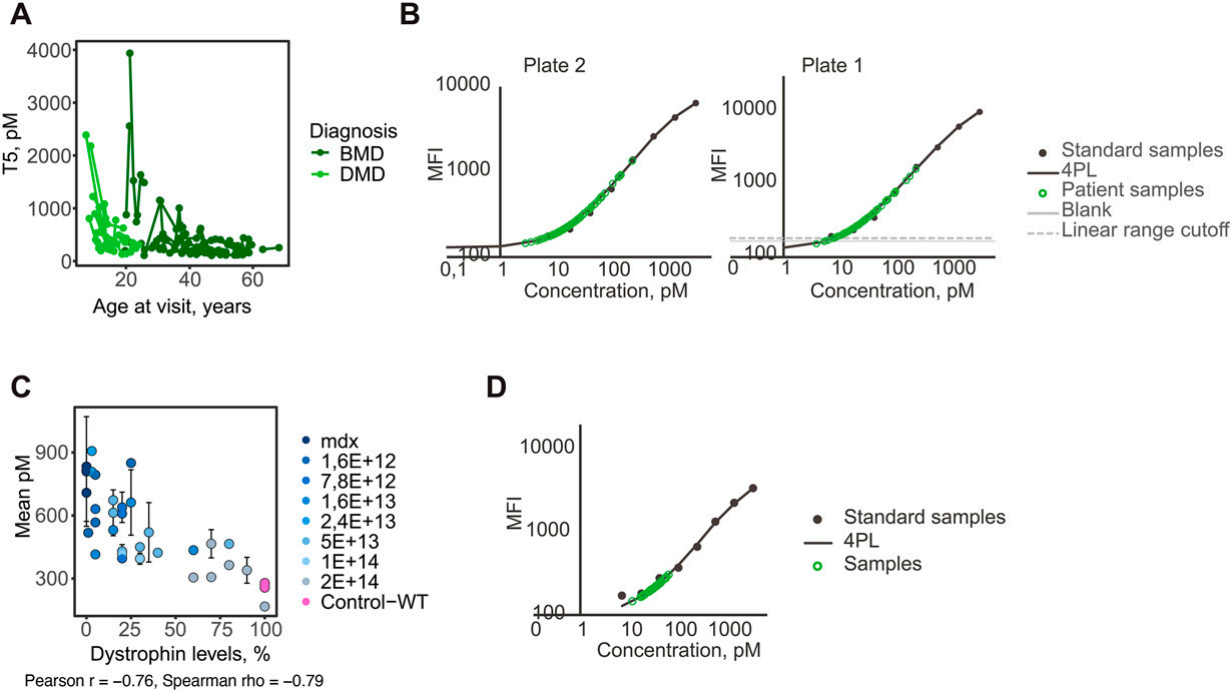
The optimised T5 self-sandwich immunoassay was used to quantify TTN fragment in serum from DMD and BMD patients and treated mdx mice. (a) Concentration of T5 titin fragment in DMD and BMD patients in pM. Samples from the same patient are connected. (b) Standard curves from analysis of DMD/BMD cohort (divided over two assay plates), with patient samples overlayed (green). Approximately half of the samples were close to the lower detection limit of the assay. (c) Concentration of T5 titin fragment in *mdx* mice treated with micro-dystrophin therapy correlated with dystrophin/micro-dystrophin levels in quadriceps muscle. Samples were measured in duplicate. (d) Standard curve for mdx cohort with mouse samples overlayed (green). All samples were close to detection limit of assay, which can explain the high variation observed between replicates.

The developed sandwich assay was used to quantify a 100 kDa fragment of TTN in the longitudinal patient cohort (Figure 2(a)). TTN concentrations ranged between 130 and 2,390 pM (median of 384 pM) in DMD patients and 110 and 3,970 pM (median of 271 pM) in BMD patients. However, 6 DMD samples and 31 BMD samples had TTN levels close to the lower detection limit of the assay (Figure 2(b)), rendering these measurements less reliable. Quantification of TTN was performed using diluted (1:12.5) patient serum samples, which allows for the adjustment of a wider quantification range. Nevertheless, this points to a large dynamic range for this TTN fragment in both DMD and BMD patients. Furthermore, the same assay proved to be translatable to mouse plasma samples (Figure 2(c) and (d)), where TTN was quantified between 250 and 990 pM. The TTN serum concentrations had a Pearson correlation of -0.76 (*p* = 1.53e−08) and Spearman ρ correlation of -0.79 (*p* = 1.53e−08) with micro-dystrophin expression in mdx quadriceps muscle, estimated as the percentage of muscle fibres in whole muscles exhibiting positive anti-dystrophin antibody staining, as had been previously observed for the HPA030048 antibody.

**Figure 3.**
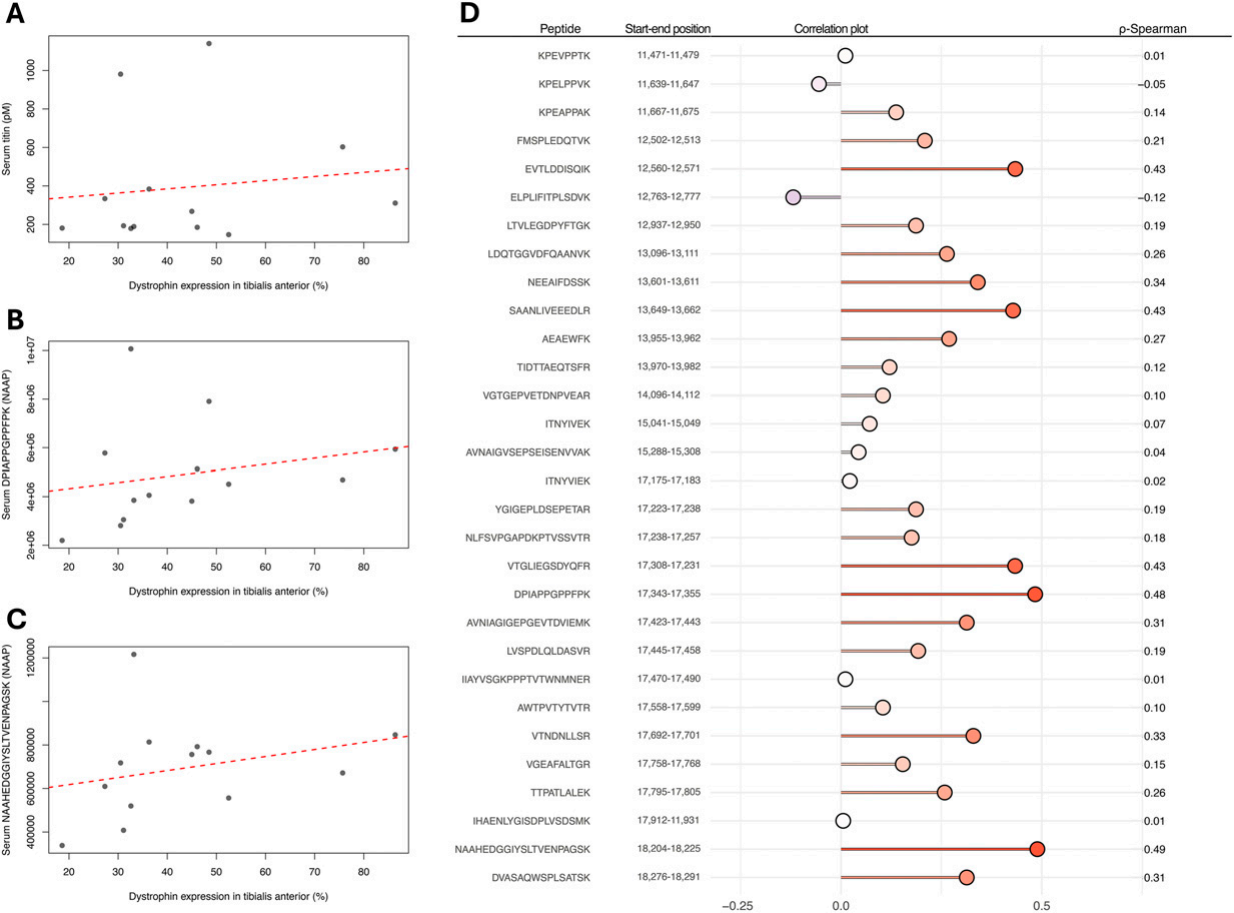
The correlation between TTN serum levels and BMD patients’ expression of dystrophin in TA. Scatter plots representing the correlation between dystrophin expression in muscle and serum TTN concentrations (a) measured using the T5 sandwich immunoassay and the serum TTN peptides (b) DPIAPPGPPFPK and (c) NAAHEDGGIYSLTVENPAGSK measured by mass spectrometry. Dot-plot (d) representing the correlation of the different serum TTN peptides and dystrophin expression in TA from BMD patients.

To explore if serum TTN levels quantified using the immunoassays are associated with dystrophin expression in muscle, as observed in mdx mice treated with the micro-dystrophin therapy, we estimated the correlation in BMD patients who have donated muscle biopsies. Serum TTN concentrations (Figure 3(a)) with dystrophin expression in TA, had a Spearman correlation value of 0.31. Similar correlation analysis between the quantified serum peptides and dystrophin expression showed large variation as visualised by the bar-style dot-plot (Figure 3(d)). Peptides detected within the region recognised by the antibodies used to construct the sandwich immunoassay had Spearman correlation values between 0.02 and 0.19. The dot-plot reveals variations and possible “r-hotspots” with the DPIAPPGPPFPK (Figure 3(b)) and NAAHEDGGIYSLTVENPAGSK (Figure 3(c)) peptides showing the highest correlation. However, none of these correlations reached statistical significance after adjustment (Supplemental Table S5).

## 4. Discussion

Titin (TTN) has increasingly gained interest as a biomarker candidate for Duchenne muscular dystrophy since the discovery of TTN fragments in urine.^14^ High titin levels in urine were reported to reflect inability to walk and weaker grip strength in BMD patients.^39^ While collection of urine is much less invasive than serum, urine samples have a greater within-patient variability than serum or plasma due to differences in patient hydration, urine water content and flow rate during sample collection,^40^ which needs to be accurately controlled in immunoassays. In addition, assuming that TTN proteolytic fragments in serum are derived from dystrophic striated muscles, only a subset of these fragments will be able to pass through the kidneys into urine. More recently, plasma TTN levels in mdx mice have been reported to correlate with dystrophin expression in skeletal muscle subsequent to treatment with micro-dystrophin restoring gene therapy.^8^ Serum or plasma would be an optimal matrix for TTN characterisation and biomarker development. In this study, based on mass spectrometry results regarding the presence of different TTN peptides in serum, we developed a protein quantification assay to measure serum TTN concentrations in samples from DMD and BMD as well as *mdx* mice. Six antibodies were tested for their performance and ability to not only detect the correct protein but also confirm, as previously reported.^8^ The developed assay detected TTN levels that significantly correlated with restoration of dystrophin expression in mdx mice and with disease severity using natural history studies in DMD and BMD patients, but did not show a significant correlation with dystrophin expression in BMD patients.

While bottom-up tandem mass spectrometry is an attractive strategy for proteomics analysis of complex samples, such as serum, the trypsin digestion of proteins prior to MS analysis removes the link between protein size and detected peptides. Furthermore, peptides overlapping with protease cleavage sites will not be detectable since they are missing from the *in silico*-generated peptide database. By combining antibodies with bottom-up tandem mass spectrometry, we could identify a fragment of TTN with an approximate size of 100 kDa in serum. Previously, serum levels of TTN have been detected using Western blot,^17^ mass spectrometry^41,42^ and affinity-proteomics,^8,43^ but the size and sequence of detected protein parts are poorly understood. In this study, we used results from an SRM tandem mass spectrometry experiment on a longitudinal patient serum cohort to guide the development of six new monospecific anti-TTN antibodies raised against six 200 amino acid-long stretches of TTN with low homology to other human proteins. We demonstrated that the antibody T5, raised against amino acid sequence 17,105–17,304 of soleus TTN, and confirmed previously reported characteristics: (1) TTN levels were higher in DMD patients, BMD patients compared to healthy controls with FDR of 6.28E-04 and 4.25E–8 respectively (2) the time-dependent decline of serum TTN over time was steeper in DMD patients compared to BMD patients, with a significant adjusted P-value of 3.25E–02 confirming the association with disease severity.^8,44^ However, the T5 antibody does not clearly differentiate between DMD and BMD patients. Previous reports comparing age-matched DMD and BMD patients revealed that differences in titin levels were observed between DMD and BMD patients within the 3–10 and 16–33-year age groups, but not 10-15 years.^44^ The samples used in this study were collected from DMD and BMD patients outside these age ranges. Only 16 samples were collected within the same age range from both DMD and BMD patients, making a comparison between the concentration of serum titin in DMD and BMD patients inconclusive. Furthermore, the limited number of not only patient samples but also healthy age-matched controls limits the interpretation of our results. Currently, there are no universal strategies to facilitate the collection of samples from patients and age-matched individuals. A possible future strategy is to include healthy family members who are willing to participate in clinical studies.

The antibody used to develop the immunoassay enabled the identification of 2 proteins of >250 kDa and 39 kDa in human skeletal muscle lysate (Figure 1(b)), showing low cross-reactivity to other proteins. In contrast, the 100 kDa fragment recognised in the serum of patients with DMD and BMD, but not in healthy controls, is most likely a smaller TTN fragment not detectable in skeletal muscle lysate. In-gel digest LC-MS/MS confirmed that the fragment recognised by the T5 antibody is derived from titin. Furthermore, a quantitative sandwich immunoassay was developed towards aa 17,105–17,304, which could discriminate patients with different levels of dystrophin expression in both humans and mice. Showing that an antibody binds selectively to a specific protein in a specific context-of-use is a challenging task. The sheer size of TTN prevents recombinant production of the full-length protein, and the hypothesis that TTN leaks into the blood circulation as degradation products of varying sizes adds its own layer of complexity. By combining Western blot, in-gel digest mass spectrometry and immunoassays, we can show that the T5 antibody binds TTN in our application. It was reassuring that T5 only detected one band of consistent size in all patient serum samples, and with sizes considerably larger than the average human serum protein. Only one patient serum sample contained an additional band of >250 kDa. In mdx mice serum TTN concentrations, estimated with the developed sandwich immunoassay, correlate with the restoration of dystrophin expression in skeletal muscle in response to the gene therapy, confirming previous reports.^8^ Importantly, this fragment most likely reflects muscle injury rather than dystrophin abundance itself. The TTN plasma levels had a strong negative correlation with the percentage of dystrophin-expressing muscle fibers despite certain limitations. The dystrophin quantification method reflects the overall presence of dystrophin in muscle cross-sections rather than dystrophin concentration. Furthermore, untreated mdx mice are assumed to have no dystrophin expression and WT mice 100%. Quantification of muscle dystrophin using alternative absolute quantification methods can increase the accuracy of the correlation with plasma TTN levels and promote the use of the assay in preclinical studies. Quantification of TTN using the T5 sandwich immunoassay confirms previous results obtained with the antibody HPA030048, supporting the hypothesis that T5 recognises TTN. Due to the lack of samples from clinical trials on AAV9-CK8-μDys5 gene therapy, we used samples from DMD and BMD natural history studies to confirm that quantification of TTN is achievable. Serum TTN was successfully quantified in the samples analysed, but the assay identified samples with TTN levels close to the limit of detection. Due to the low sample requirement in a clinical set-up, the assay can be performed in replicates to ensure accuracy. Alternatively, the assay may benefit from a signal amplification approach like the Binding Oligo Ladder Detection method, the Exazym^**®**^ (CAVIDI), to amplify the signal. The BMD sample collection was selected to test the TTN assay as it represents a milder phenotype similar to the one expected to arise when DMD patients are treated with dystrophin-restoring therapies. The correlation with dystrophin expression in TA was low. Interestingly, although not significant, serum titin levels quantified with the sandwich immunoassay had a slightly higher correlation with the dystrophin expression in TA of BMD patients, compared to the peptides detected by mass spectrometry within the same protein fragment. Importantly, reports show that dystrophin expression in skeletal muscle does not change over time in the BMD population.^4^

Recent research has highlighted the potential of titin (TTN) as a biomarker for DMD. In this study, a protein quantification assay was developed to measure serum TTN concentrations in samples from DMD and BMD patients, as well as mdx mice. The T5 antibody identified a significant TTN fragment in the serum of DMD and BMD patients, which was not present in healthy controls. The developed TTN quantification assay is suitable for monitoring the outcome of dystrophin-restoring therapies in the mdx mouse. If dystrophin quantification using the assay is further optimised in samples from DMD patients and the correlation with dystrophin expression subsequent to treatment with dystrophin-restoring therapies is confirmed, the assay offers a less invasive approach to estimate treatment outcome, compared to the semi-quantitative analysis of dystrophin in muscle biopsies. This work demonstrated a correlation between serum TTN levels and dystrophin expression in skeletal muscle following gene therapy in mdx mice, indicating the promising role of TTN in evaluating disease progression and treatment response if translated to DMD patients. Future confirmatory studies, including samples from treated DMD patients with information regarding dystrophin expression in skeletal muscle, are, however, warranted.

## Supplemental material

Supplemental material - Quantification of a serum titin fragment reflects dystrophin restoration in mdx mice and disease severity in patients with dystrophinopathies Supplemental material for Quantification of a serum titin fragment reflects dystrophin restoration in mdx mice and disease severity in patients with dystrophinopathies by Camilla Johansson, Jessica F. Boehler, Kristy. Brown, Albert Jiménez-Requena, Andreas Hober, Fredrik Edfors, Åsa Sivertsson, Gabriella Hober, Zaïda Koeks, Nienke M. van de Velde, Mathias Uhlén, Hanna Tegel, Erik H. Niks, Pietro Spitali and Cristina Al-Khalili Szigyarto in Journal of Neuromuscular Diseases.

## Data Availability

MS data can be accessed through https://panoramaweb.org/r5jRCR.url, whereas the rest of the data can be obtained upon request.
