# Supplementary material for: Quantification of a serum titin fragment reflects dystrophin restoration in mdx mice and disease severity in patients with dystrophinopathies
Source: J Neuromuscul Dis. 2026 May 20:22143602261453984. Online ahead of print. doi: 10.1177/22143602261453984 (PMC13438847; doi:10.1177/22143602261453984)
Supplement: Supplementary file 1 [file sj-zip-1-jnd-10.1177_22143602261453984.zip › Supporting_information_TTN_v9.pdf]

Supporting information for

Development of a titin quantification immunoassay for monitoring micro-dystrophin therapeutic response

Camilla Johansson[1], Jessica F. Boehler[2], Kristy J. Brown[3], Albert Jimenéz Requena[1], Andreas Hober[4], Fredrik Edfors[4], Åsa Sivertsson[4], Gabriella Hober[1], Zaïda Koeks[5], Nienke M. van de Velde[5], Mathias Uhlén[4], Hanna Tegel[1], Erik H. Niks[5], Pietro Spitali[6] and Cristina Al-Khalili Szigyarto[1,4*]

1. Department of Protein Science, School of Chemistry, Biology and Health, KTH Royal Institute of Technology, Stockholm, Sweden.
2. Solid Biosciences Inc., 500 Rutherford Ave., Charlestown, MA, United States.
3. Somite Therapeutics, 40 Guest Street, Brighton, MA 01235, United States.
4. Science for Life Laboratory, School of Chemistry, Biology and Health, KTH Royal Institute of Technology, Stockholm, Sweden.
5. Department of Neurology, Leiden University Medical Center, Leiden, The Netherlands.
6. Department of Human Genetics, Leiden University Medical Center, Leiden, The Netherlands.

* Corresponding author
Cristina Al-Khalili Szigyarto,
Department of Protein Science, School of Chemistry, Biology and Health, KTH Royal Institute of Technology, Stockholm, Sweden,
Phone: +4687909893,
E-mail: caks@kth.se

Figures

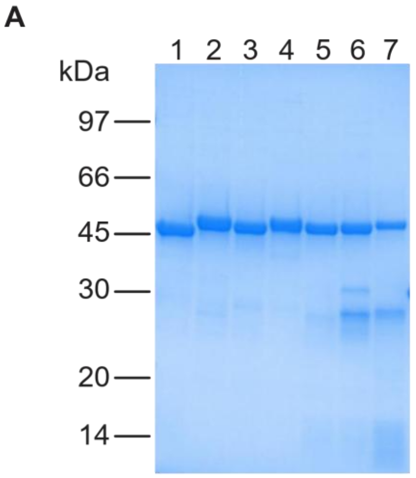

B

#	aa sequence	Comment
1	13,691 – 13,890	OK
2	13,944 – 14,143	OK
3	15,152 – 15,351	OK
4	15,387 – 15,586	OK
5	17,105 – 17,304	OK
6	17,584 – 17,783	Fail, low purity
7	17,788 – 17,987	OK

Figure S1. Production of PrESTs for antibody production. A. SDS-PAGE for PrEST purity. **B.** TTN amino acid sequences and purity decision based on gel. Numbers correspond to lanes on gel. Lane 6 failed the purity assessment and was not sent for immunisation.

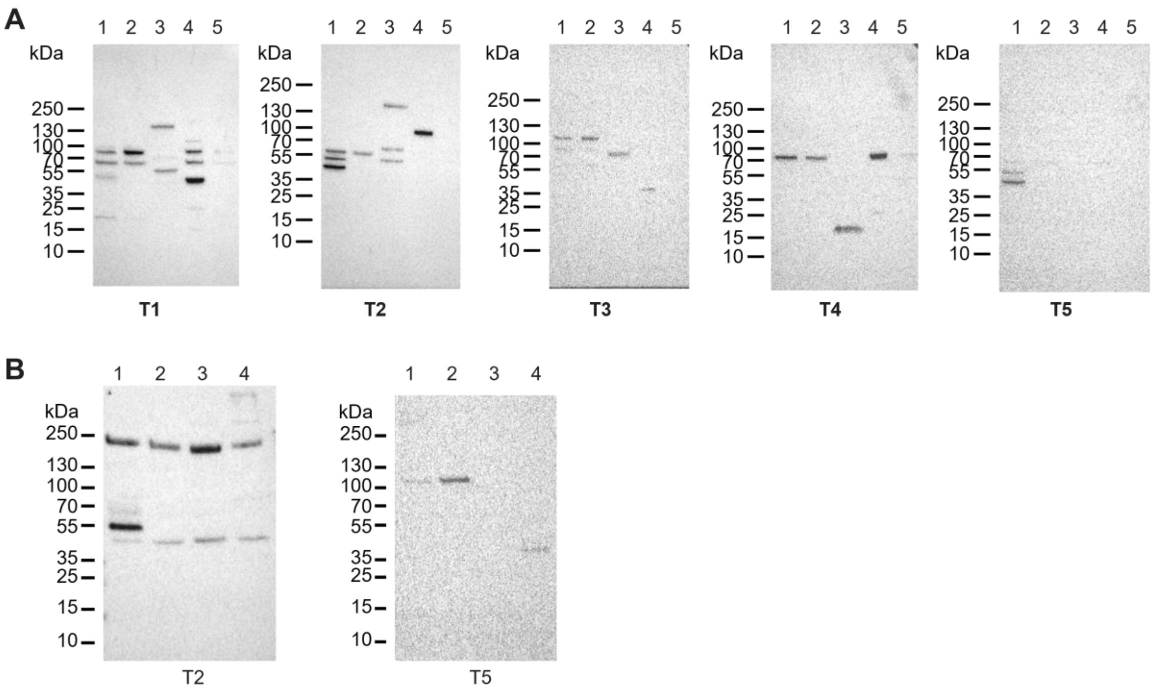

Figure S2. Binding evaluation of new antibodies using Western Blot. A. Cross-binding assessment of antibodies using a standard panel of samples. 1: RT4 cell lysate. 2: U-251 MG cell lysate. 3: Human plasma. 4: Liver. 5: Tonsils. **B.** Repetition of Western blot using patient serum samples for antibodies T2 and T5. 1: DMD pool. 2: BMD. 3: Healthy control pool. 4: normal human serum (31876, Invitrogen).

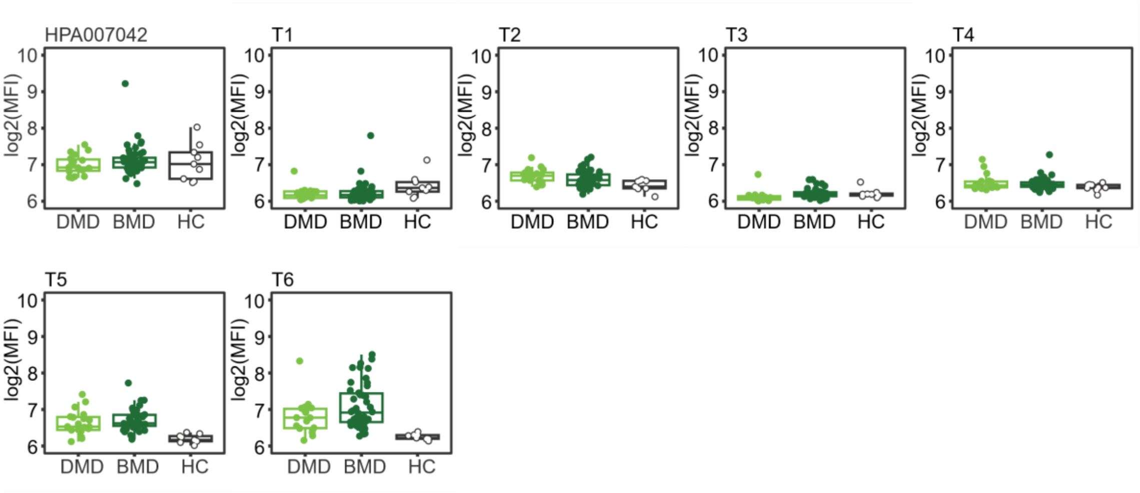

Figure S3. All anti-TTN antibodies were tested on a longitudinal serum cohort with DMD/BMD patients and healthy controls using antibody suspension bead array technology [2]. Boxplots showing difference of TTN fragments for each diagnosis. Plots only contain samples from first visit, one sample per patient.

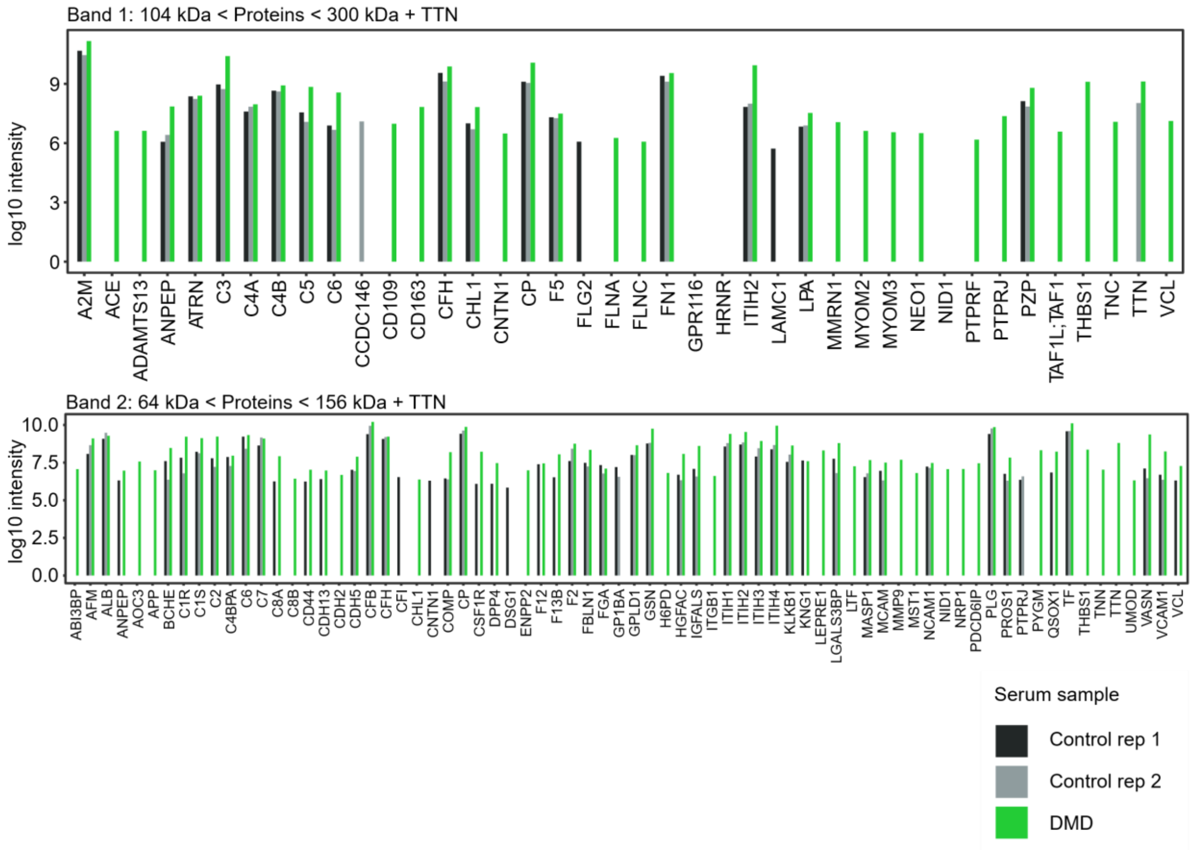

Figure S4. Intensities for proteins identified in each excised band for in-gel digest LC-MS/MS. Only proteins with molecular weight ± 20% of the excised regions are included in each figure. Keratins are excluded from the figure for band 2. Band 1 between 130 and 250 kDa and band 2 between 70 and 130 kDa.

A.

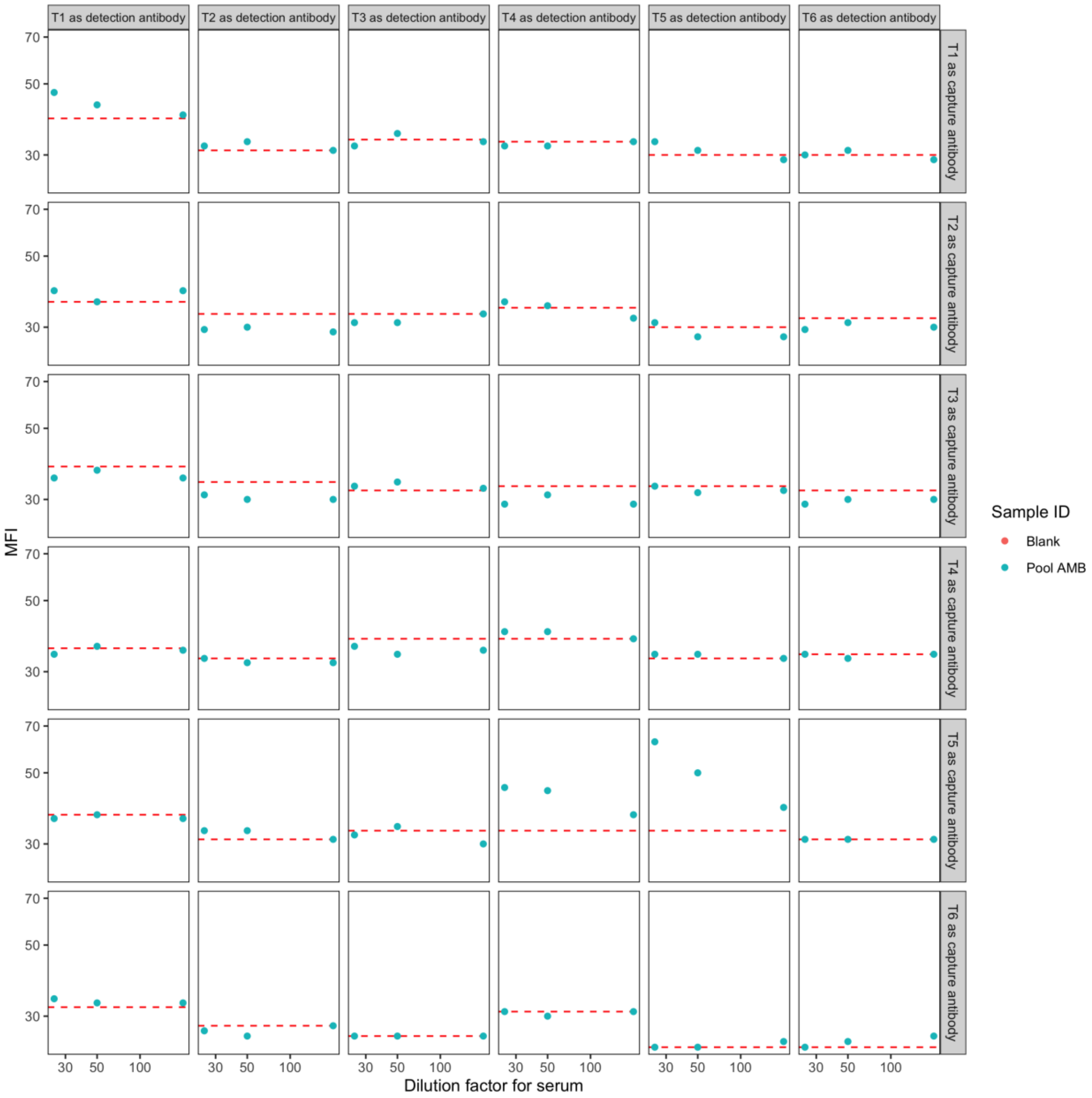
T1 as detection antibody
T2 as detection antibody
T3 as detection antibody
T4 as detection antibody
T5 as detection antibody
T6 as detection antibody
T1 as capture antibody
T2 as capture antibody
T3 as capture antibody
T4 as capture antibody
T5 as capture antibody
T6 as capture antibody
MFI
70
50
30
30
50
100
Dilution factor for serum
Sample ID
Blank
Pool AMB

B.

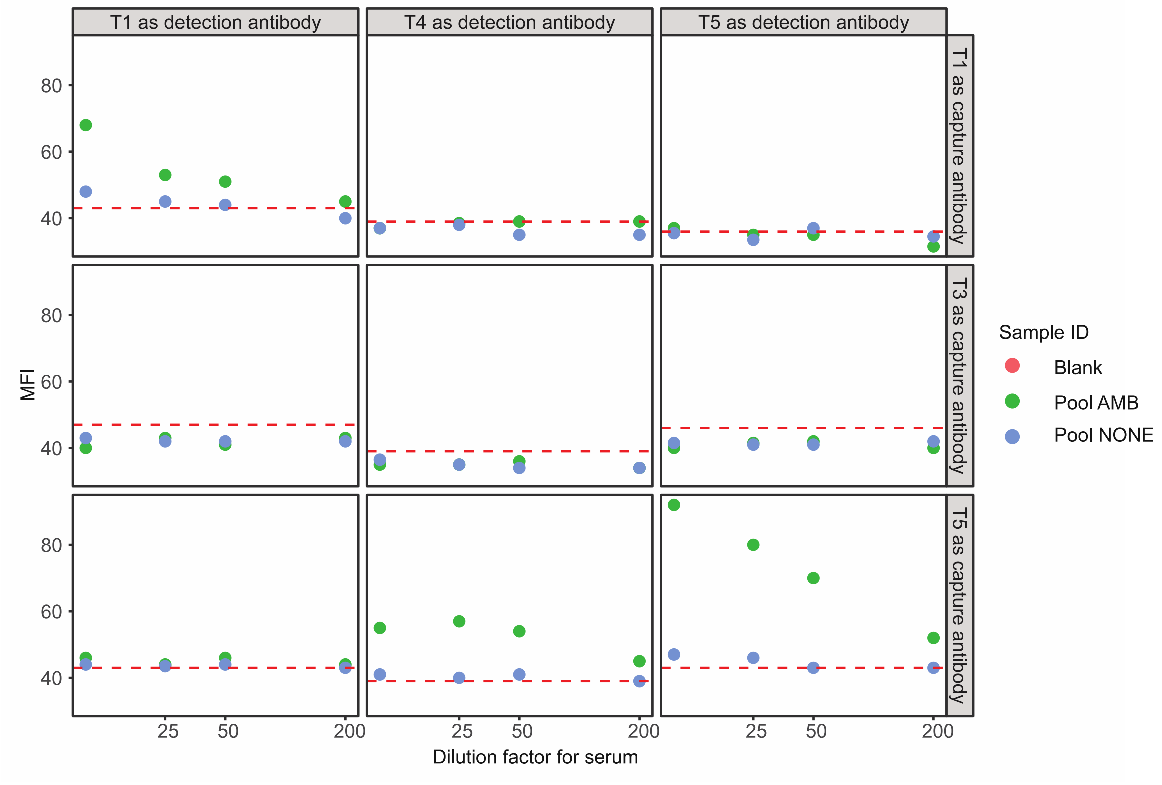

Figure S5. Antibody pair screening for sandwich immunoassay. A. T5 and T1 self-sandwich had a 2-fold and 1.2-fold higher signal compared to the background in serum at 1:25 from ambulant DMD patients (Pool AMB). The sandwich with T5 as capture antibody and T4 as detection antibody had 1.3-fold higher signals than the background. B. T1 and T5 self-sandwich and the T5-T4 sandwich showed dose-dependent signals with higher signals in serum from ambulant DMD patients compared to non-ambulant patients (Pool NONE). A pooled sample from non-ambulant DMD patients (Pool NONE) did not show a signal above background for any antibody combination.

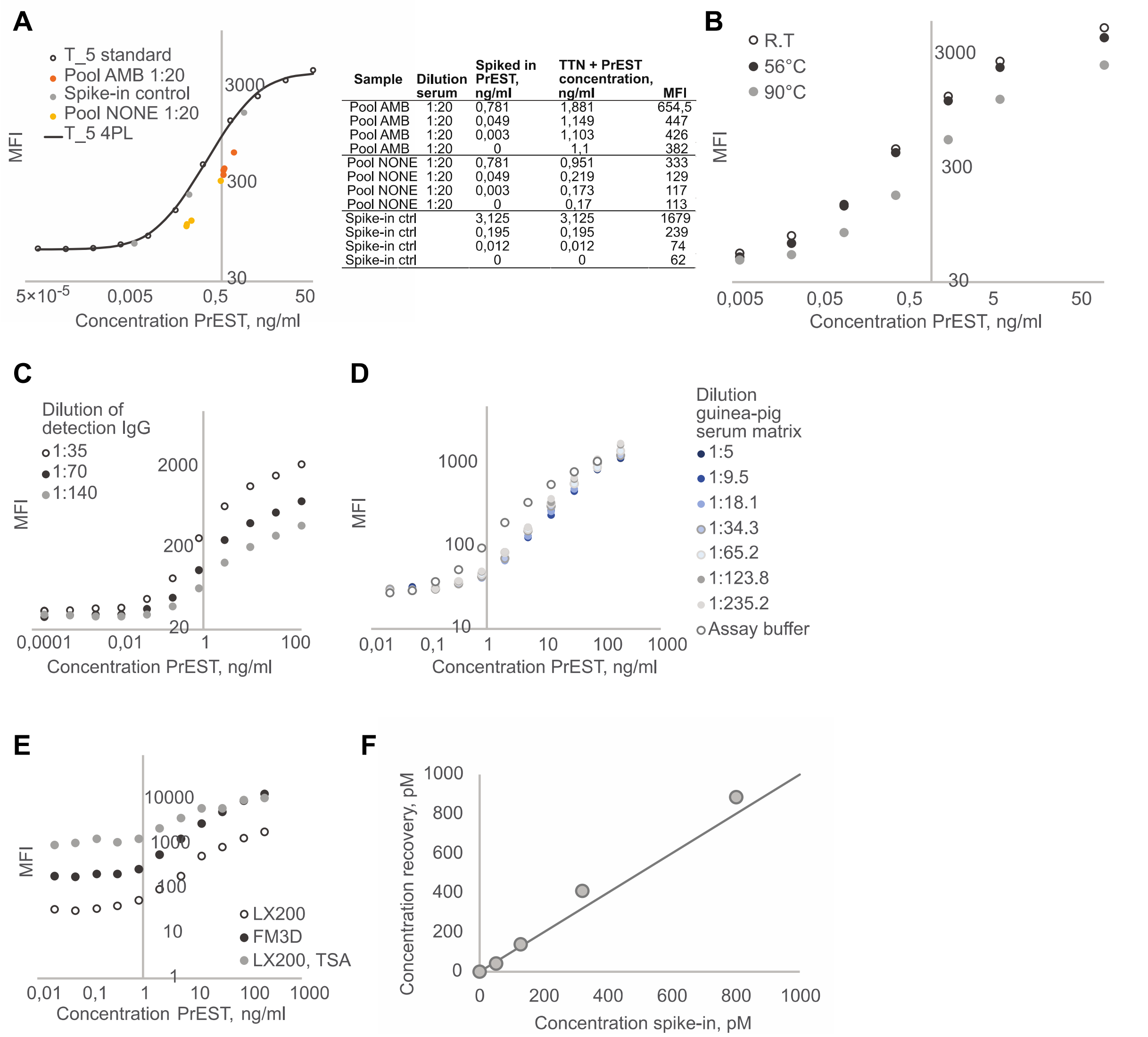

Sample	Dilution serum	Spiked in PrEST, ng/ml	TTN + PrEST concentration, ng/ml	MFI
Pool AMB	1:20	0,781	1,881	654,5
Pool AMB	1:20	0,049	1,149	447
Pool AMB	1:20	0,003	1,103	426
Pool AMB	1:20	0	1,1	382
Pool NONE	1:20	0,781	0,951	333
Pool NONE	1:20	0,049	0,219	129
Pool NONE	1:20	0,003	0,173	117
Pool NONE	1:20	0	0,17	113
Spike-in ctrl		3,125	3,125	1679
Spike-in ctrl		0,195	0,195	239
Spike-in ctrl		0,012	0,012	74
Spike-in ctrl		0	0	62

Figure S6. Immunoassay development for T5 self-sandwich. A. A standard curve was created using the immunogen (T5 PrEST) for the T5 antibody. Spike-ins of T5 PrEST were performed in serum pools of ambulant DMD (Pool AMB) and non-ambulant DMD (Pool NONE) samples diluted 1:20. The spike-in experiment showed a possible matrix effect. **B.** The effect of heat-inactivation on detection range was assessed. Heat-inactivation at 56°C for 30 min is normally performed [2] to inactivate residual complement activity. Experiment showed no significant difference between no heat-inactivation and inactivation at 56°C, but reduced detection range (aggregation) when inactivating at 90°C. **C.** Optimizing dilution of detection antibody showed that the detection antibody needs to be diluted no more than 1:35 to maximise detection range. **D.** To mimic the matrix effect observed in fig 7A, standard curve assay buffer was spiked with different concentrations of guinea-pig serum (ranging from 1:5 to 1:235). A concentration-dependent matrix effect was observed that mimicked what was observed in patient samples. **E.** The immunoassay was tested at two different instruments from Luminex, the LX200 with a dynamic range ≥3.5 logs of signal and the FlexMap3D with a dynamic range ≥4.5 logs of signal. Signal amplification using Tyramide Signal Amplification (TSA) was also tested. TSA could improve signal amplitude, but also amplified background. **F.** Optimal test parameters were selected (56°C heat denaturation, 1:35 detection antibody dilution, guinea-pig serum spike-ins in standard curve on FM3D instrument). Spike-in recovery of PrEST antigen in pooled DMD serum showed good recovery (< 20 % CV).

Tables

Table S1. **Patient samples included in the study comprise male DMD, BMD and healthy controls**

Patient group	Number of individuals	Number of samples	Median age (Min and Max age)	Dystrophin expression (SD)
**BMD**	34	100	44.3 (19.8-68.2 years)	-
**BMD w. biopsy**	13	44	43,5 (14.8-57.6 years)	43.3% (19.3)
**DMD**	19	49	14.3 (7.4-24.0 years)	-
**Healthy controls**	9	9	11.0 (6.3-50.5 years)	-

Table S2. Identified TTN peptides.

Peptide	aa start	aa end	Ensembl ID	Median normalised area
ELPLIFITPLSDVK	*14,405*	*14,418*	*ENSP00000467141*	*43,784*
KPEVPPTK	*13,063*	*13,070*	*ENSP00000467141*	*3,333,400*
KPEVPPTK	*12,700*	*12,707*	*ENSP00000467141*	*3,333,400*
KPEVPPTK	*12,449*	*12,456*	*ENSP00000467141*	*3,333,400*
KPEVPPTK	*12,198*	*12,205*	*ENSP00000467141*	*3,333,400*
KPELPPVK	*13,147*	*13,154*	*ENSP00000467141*	*109,615*
TIDTTAEQTSFR	*15,612*	*15,623*	*ENSP00000467141*	*231,435*
DPIAPPGPPFPK	*17,343*	*17,355*	*ENSP00000467141*	*3,307,000*
SAANLIVEEEDLR	*15,291*	*15,303*	*ENSP00000467141*	*361,775*
TTPATLALEK	*19,437*	*19,446*	*ENSP00000467141*	*37,059,000*
NEEAIFDSSK	*15,243*	*15,252*	*ENSP00000467141*	*47,593,500*
DVASAQWSPLSATSK	*19,918*	*19,932*	*ENSP00000467141*	*12,312,500*
VTNDNLLSR	*19,334*	*19,342*	*ENSP00000467141*	*16,084,500*
AEAEWFK	*15,597*	*15,603*	*ENSP00000467141*	*576,410,000*
AVNIAGIGEPGEVTDVIEMK	*19,065*	*19,084*	*ENSP00000467141*	*2,419,750*
AVNAAGVSKPSATVGPVTVK	*18,661*	*18,680*	*ENSP00000467141*	*9,620,450*
LDQTGGVDFQAANVK	*14,738*	*14,752*	*ENSP00000467141*	*556,045*
LVSPDLQLDASVR	*19,087*	*19,099*	*ENSP00000467141*	*1,334,550*
NLFSVPGAPDKPTVSSVTR	*18,880*	*18,898*	*ENSP00000467141*	*21,656,500*
AWTPVTYTVTR	*19,230*	*19,240*	*ENSP00000467141*	*3,157,900*
FMSPLEDQTVK	*14,144*	*14,154*	*ENSP00000467141*	*6,775,150*
ITNYIVEK	*33,721*	*33,728*	*ENSP00000467141*	*2,877,150*
ITNYIVEK	*31,341*	*31,348*	*ENSP00000467141*	*2,877,150*
ITNYIVEK	*27,798*	*27,805*	*ENSP00000467141*	*2,877,150*
ITNYIVEK	*23,467*	*23,474*	*ENSP00000467141*	*2,877,150*
ITNYIVEK	*21,592*	*21,599*	*ENSP00000467141*	*2,877,150*
ITNYIVEK	*16,683*	*16,690*	*ENSP00000467141*	*2,877,150*
ITNYVIEK	*19,216*	*19,223*	*ENSP00000467141*	*3,241,150*
ITNYVIEK	*18,817*	*18,824*	*ENSP00000467141*	*3,241,150*
AVNAIGVSEPSEISENVVAK	*16,930*	*16,949*	*ENSP00000467141*	*518,315,000*
LTVLEGDPYFTGK	*14,579*	*14,591*	*ENSP00000467141*	*8,557,150*
NAAHEDGGIYSLTVENPAGSK	*19,846*	*19,866*	*ENSP00000467141*	*611,765*
YGIGEPLDSEPETAR	*18,865*	*18,879*	*ENSP00000467141*	*1,497,300*
KPEAPPAK	*13,175*	*13,182*	*ENSP00000467141*	*8,861,100*
EVTLDDISQIK	*14,202*	*14,212*	*ENSP00000467141*	*385,180*
VTGLIEGSDYQFR	*18,950*	*18,962*	*ENSP00000467141*	*87,759*
IIAYVSGKPPPTVTWNMNER	*19,112*	*19,131*	*ENSP00000467141*	*1,312,550*
VGEAFALTGR	*19,400*	*19,409*	*ENSP00000467141*	*345,320*
IHAENLYGISDPLVSDSMK	*19,554*	*19,572*	*ENSP00000467141*	*6,861,800*
VGTGEPVETDNPVEAR	*15,738*	*15,753*	*ENSP00000467141*	*43,329,000*
